# Drug-Induced Angioedema Without Urticaria: A Case Report

**DOI:** 10.7759/cureus.64452

**Published:** 2024-07-13

**Authors:** Shrinivas R Raikar, Sreeraj G, Sneha Sneha, Janarthanan R

**Affiliations:** 1 Pharmacology, Bijapur Lingayat District Educational University (BLDE Deemed to be University) Shri B. M. Patil Medical College Hospital and Research Centre, Vijayapura, IND

**Keywords:** adverse drug reactions, non-steroidal anti-inflammatory drugs, leukotrienes, angioedema, diclofenac

## Abstract

Non-steroidal anti-inflammatory drugs (NSAIDs) are widely prescribed for various conditions but are associated with numerous adverse drug reactions (ADRs). Understanding these ADRs is necessary to reduce morbidity and mortality. NSAID-induced angioedema, although rare, can be life-threatening and is often due to increased leukotriene production from COX pathway inhibition. Mast cells and basophil degranulation play vital roles in its pathogenesis. Prompt recognition and immediate cessation of the culprit drug, along with the administration of corticosteroids and antihistamines, are essential. Here, we report a case of angioedema caused by diclofenac administration, which needs prompt vigilance and a rapid therapeutic response.

## Introduction

Angioedema is characterized by localized swelling of the skin and mucous membranes that tends to occur predominantly in the face, lips, tongue, throat, extremities, and genitalia. The major predisposing factors are food allergies, environmental allergens, and medications such as non-steroidal anti-inflammatory drugs (NSAIDs), angiotensin-converting enzyme inhibitors, angiotensin receptor blockers, penicillins, fluoroquinolones, and sulfonamides. Although they offer therapeutic benefits, NSAIDs can trigger rare but serious life-threatening angioedema. Diclofenac, an extensively used NSAID, is associated with angioedema and other adverse drug reactions (ADRs). The primary mechanism involves COX inhibition by NSAIDs, which leads to reduced prostaglandin synthesis and overproduction of leukotrienes, which in turn increase vascular permeability, ultimately causing angioedema [1].

## Case presentation

**Figure 1:**
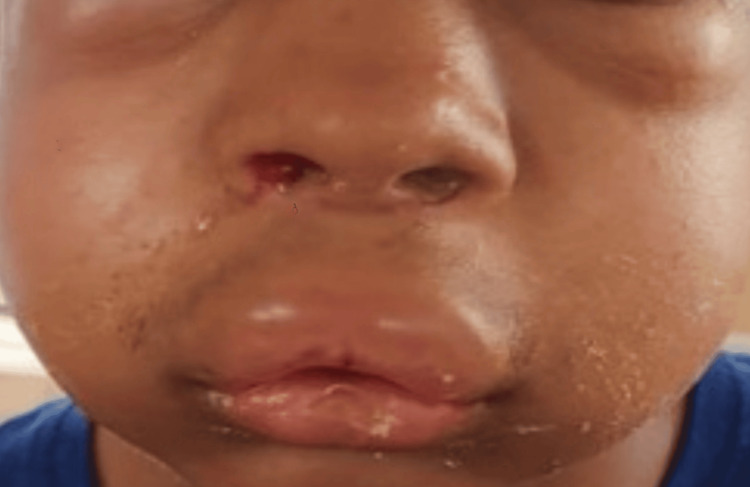
Diclofenac-induced hypersensitivity reaction - angioedema

A 16-year-old male patient presented with bilateral nasal obstruction persisting for two years. The patient was diagnosed with bilateral deviated nasal septum, sinusitis, and hypertrophy of the right inferior turbinate. The patient had no history of drug allergies in the past. The patient underwent septoplasty and reduction of the right inferior turbinate under general anesthesia without any complications. Postoperatively, he was shifted to the ward and started on cefotaxime injection (IV) infusion 1 gm in 100 ml normal saline BD, pantoprazole injection (IV) 40 mg OD, and diclofenac injection sodium 75 mg (IV) infusion in 100 ml normal saline BD. However, he developed an ADR characterized by generalized facial edema, notably around the lips and periorbital region, which was not associated with urticaria or rash (Figure 1). The drug diclofenac was suspected and discontinued immediately. The patient was treated with corticosteroids and antihistamines. Within six hours, the swelling subsided, leading to a complete recovery thereafter. Using Naranjo’s algorithm, the assessment categorized the reaction as “possible.” As per the Hartwig-Siegel scale, the severity of the reaction was classified as "moderate,” while the modified Schumock Thornton Scale indicated the reaction was “non-preventable.”

## Discussion

Angioedema, a common side effect of widely used drugs, is usually self-limiting but can pose a risk of life-threatening respiratory obstruction. It manifests suddenly as non-pitting erythematous swelling of the skin and mucous membranes, usually without accompanying urticaria. This happens due to increased vascular permeability caused by the activation of mast cells, basophils, and histamine release. Other mediators, like leukotrienes and prostaglandins, may also contribute to this condition.

Several studies reveal NSAIDs and antibiotics as the key medications involved in drug-induced angioedema, which often occurs in the absence of urticaria. The cause of NSAID-induced angioedema may involve immunological or non-immunological mechanisms, possibly associated with increased leukotriene production due to the inhibition of the cyclooxygenase pathway [2].

Non-specific COX inhibitors such as ibuprofen and aspirin are commonly associated with NSAID-induced angioedema, although selective and preferential COX-2 inhibitors like diclofenac and meloxicam can also trigger these reactions. Selective COX-2 inhibitors are less likely to cause NSAID-induced angioedema compared to non-selective COX inhibitors. Non-selective COX inhibitors block both COX-1 and COX-2 enzymes, leading to reduced prostaglandin synthesis and increased leukotriene production. Selective COX-2 inhibitors target COX-2, thereby maintaining some level of prostaglandin production and decreasing the leukotriene-mediated inflammatory response. Angiotensin-converting enzyme inhibitors, which are known to worsen the condition of angioedema, should also not be taken along with NSAIDs [3]. In treating NSAID-induced angioedema, it is necessary to identify the offending NSAID early and withdraw it immediately. Management plans depend on the extent of severity, ranging from corticosteroids and antihistamines for mild cases to potential interventions like tracheostomy or intubation for severe reactions. Most reactions subside within 48 to 72 hours post-treatment. However, the effectiveness of leukotriene receptor antagonists in preventing exacerbations due to NSAID-induced angioedema is still debated in recent studies.

To avoid the recurrence of such incidents, further treatment rests on the education of the patient regarding what he or she needs to do to avoid the triggering factors. Patients sensitive to diclofenac may have other respiratory allergies, and, therefore, there is a possibility of cross-sensitivity with other NSAIDs, hence the need for careful choice of other drugs to be prescribed. While selective COX-2 inhibitors may be considered in diclofenac-intolerant patients, further investigation is needed to establish their safety and efficacy in this context. This case was reported to the Adverse Drug Monitoring Centre PVPI, BLDE (DU), Shri B. M. Patil Medical College Hospital and Research Centre, Vijayapura. The worldwide unique ID is IN-IPC-300937906 for this case under the Indian Pharmacopoeia Commission.

## Conclusions

ADRs, notably angioedema, can occur during diclofenac therapy, necessitating rigorous monitoring and immediate intervention. Diclofenac has been associated with instances of angioedema, a condition also commonly linked to NSAIDs and antibiotics. Angioedema in general is self-limiting; however, it appears to be a life-threatening condition due to the possibility of airway compromise and death. There should be early detection and elimination of the offending agent. Treatment modalities such as adrenaline, corticosteroids, and antihistamines can be used appropriately to relieve the symptoms. Patient education regarding the identification and avoidance of the offending drug is paramount, and healthcare providers should maintain a high index of suspicion for ADRs. Further research is warranted to elucidate the mechanisms and risk factors associated with diclofenac-induced angioedema.
